# The Yeast DNA Damage Checkpoint Kinase Rad53 Targets the Exoribonuclease, Xrn1

**DOI:** 10.1534/g3.118.200767

**Published:** 2018-10-30

**Authors:** Jessica P. Lao, Katie M. Ulrich, Jeffrey R. Johnson, Billy W. Newton, Ajay A. Vashisht, James A. Wohlschlegel, Nevan J. Krogan, David P. Toczyski

**Affiliations:** * Department of Biochemistry and Biophysics, University of California, San Francisco, CA 94158; † Department of Cellular and Molecular Pharmacology, University of California, San Francisco, CA 94158; ‡ Department of Biological Chemistry, School of Medicine, University of California, Los Angeles, CA 90095

**Keywords:** DNA Damage Response, checkpoint, Xrn1, Rad53, phosphoproteomics

## Abstract

The highly conserved DNA damage response (DDR) pathway monitors the genomic integrity of the cell and protects against genotoxic stresses. The apical kinases, Mec1 and Tel1 (ATR and ATM in human, respectively), initiate the DNA damage signaling cascade through the effector kinases, Rad53 and Chk1, to regulate a variety of cellular processes including cell cycle progression, DNA damage repair, chromatin remodeling, and transcription. The DDR also regulates other cellular pathways, but direct substrates and mechanisms are still lacking. Using a mass spectrometry-based phosphoproteomic screen in *Saccharomyces cerevisiae*, we identified novel targets of Rad53, many of which are proteins that are involved in RNA metabolism. Of the 33 novel substrates identified, we verified that 12 are directly phosphorylated by Rad53 *in vitro*: Xrn1, Gcd11, Rps7b, Ded1, Cho2, Pus1, Hst1, Srv2, Set3, Snu23, Alb1, and Scp160. We further characterized Xrn1, a highly conserved 5′ exoribonuclease that functions in RNA degradation and the most enriched in our phosphoproteomics screen. Phosphorylation of Xrn1 by Rad53 does not appear to affect Xrn1’s intrinsic nuclease activity *in vitro*, but may affect its activity or specificity *in vivo*.

Cells incur DNA damage from both endogenous and exogenous sources. To ensure faithful cell division, the highly conserved DNA damage response (DDR) pathway monitors genomic integrity and protects against genotoxic stresses. Genome instability is a common characteristic of aging cells and cancer cells and components of the DDR machinery are often mutated in cancer (Jackson and Bartek 2009; Negrini *et al.* 2010; Ashworth *et al.* 2011). In *Saccharomyces cerevisiae*, DNA damage activates the sensor kinases, Mec1 and Tel1 (ATR and ATM in human, respectively) (Melo and Toczyski 2002; Ciccia and Elledge 2010; Blackford and Jackson 2017). The response is further amplified by activation of the effector kinases, Rad53 and Chk1, to regulate a variety of cellular processes including cell cycle progression, DNA damage repair, chromatin remodeling, and transcription.

The DDR induces a number of physiological changes within the cell, including changes in gene expression and protein levels. At the gene expression level, microarray-based transcriptomic analyses identified transcripts that are up- or down-regulated in a Mec1/Tel1 dependent manner (Jelinsky and Samson 1999; Gasch *et al.* 2001). In addition, proteomics analyses identified targets of ATM/ATR through the enrichment of phosphopeptides (Matsuoka *et al.* 2007; Smolka *et al.* 2007). Many of these DDR regulated-transcripts and protein targets have known roles in DNA damage repair and cell cycle regulation, but the significance of other targets has not been characterized. In addition, the DDR affects other cellular pathways for which direct targets are not known. For example, Mec1 has been shown to induce expression of genes involved in carbohydrate metabolism and reactive oxygen species (ROS) detoxification, and down regulates the expression of ribosomal protein genes in DNA damage (Gasch *et al.* 2001). Putative substrates of ATM and ATR include proteins involved in RNA modification and cell structure (Matsuoka *et al.* 2007). Several studies also reveal the involvement of ATM in insulin signaling, AKT signaling, and the pentose phosphate pathway (Khalil *et al.* 2011; Cosentino *et al.* 2011; Fraser *et al.* 2011). Thus, novel substrates of the DDR remain to be discovered.

One area of regulation that is not well understood is the direct effect of the DDR on post-transcriptional regulation of gene expression. As an intermediate between genes and proteins, altering the abundance of mRNAs would effectively affect protein levels as well. One of the key players of mRNA dynamics is Xrn1. Xrn1 is a conserved 5′-3′ exoribonuclease that preferentially degrades 5′ monophosphorylated single-stranded RNA (Jones *et al.* 2012; Nagarajan *et al.* 2013). This arises in the cell when mRNAs are decapped prior to degradation or during processing of rRNA or tRNA (Chernyakov *et al.* 2008; Whipple *et al.* 2011; Harigaya and Parker 2012; Braun *et al.* 2012; Wu and Hopper 2014). Xrn1 is a component of the cytoplasmic processing (P) bodies and stress granules that are involved in mRNA sequestration and decay, and is responsible for the majority of mRNA degradation in the cell (Stevens *et al.* 1991; Bashkirov *et al.* 1997; He *et al.* 2003; Kedersha *et al.* 2005; Newbury 2006; Lindahl *et al.* 2009). Involvement of Xrn1 in DNA damage repair comes from the observation that *xrn1∆* cells are sensitive to DNA damaging agents, but the mechanism for how this occurs is not known (Page *et al.* 1998; Manfrini *et al.* 2014).

Here we identified 33 novel substrates of Rad53 using a phosphoproteomic screen, and confirmed that Rad53 directly phosphorylates 12 of them *in vitro*. Many of these substrates are involved in mRNA and rRNA processing and turnover. Specifically, we show that Rad53 phosphorylates Xrn1 *in vivo* and *in vitro*, linking the DNA damage response to the regulation of RNA metabolism. We found that phosphorylation does not affect the nuclease activity of Xrn1. Mutations in the C terminus of Xrn1, where we identified an enrichment of Rad53 dependent phosphorylation sites, compromise its nuclease activity. While previous observations suggested that deletion of *XRN1* rendered cells DNA damage-sensitive, our data suggests that this pathway is directly regulated by the DNA damage response pathway.

## Material and Methods

### Strains

All strains used in this study are in the S288c background unless otherwise noted (Supplemental Table 1). Strains were grown in YM-1 + 2% dextrose at 30° unless otherwise noted. Standard genetic procedures of transformation and tetrad analysis were followed to construct strains. Unless otherwise specified, GFP-tagged strains came from the yeast GFP collection made by Erin O’Shea and Jonathan Weissman (Huh *et al.* 2003) and is available through Thermo Fisher Scientific.

### Kinase Over-expression system

The kinase over-expression strains were generated by promoter replacement. To generate *TEF1pr-DUN1*, the *TEF1* promoter was amplified by PCR from the genomic DNA of yeast strain ERE92, which contains a *HphMX6-TEF1pr* construct. The PCR primers included homology arms designed to insert immediately upstream of *DUN1* by yeast transformation. To generate *GAL1pr-RAD53*, we amplified the *GAL1* promoter from pFA6a-*kanMX6-pGAL1-GFP* (Addgene Plasmid #53205) by PCR with homology arms just upstream of *RAD53*. The PCR product was purified and transformed into yeast cells. To induce *GAL1pr-RAD53*, overnight yeast cultures grown in YPD were diluted in fresh media as standard practice and incubated in a shaking incubator for 4 hr at 30° until OD_600_ reaches ∼0.8. Then, cell cultures were transferred to conical tubes and centrifuged at maximum speed to form pellets. The pellets were washed twice with fresh YP media to remove glucose, and resuspended in YP + galactose (2%). The cells were returned to growth for 1 hr to induce the *GAL1* promoter. Subsequently, 4-NQO was added to the cultures to a final concentration of 2 µg/mL. After 15 min, the cells were harvested and proteins were extracted for mass spectrometry as described below.

### Mass spectrometry

Cells were grown to mid-log phase in C-lysine-arginine media, supplemented with heavy labeled lysine and arginine or unlabeled control lysine and arginine at a concentration of 0.06 mg/mL. Cell pellets were lysed in a denaturing urea buffer (8 M urea, 0.1 M Tris pH 8, 150 mM NaCl, 1 Roche mini protease inhibitor tablet without EDTA/10 mL, 10 mM sodium butyrate and 10 mM nicotinamide) in a BioSpec bead-beater. Extracts were treated with 1 M TCEP (Sigma C4706-2), then 0.5 M iodoacetamide (Sigma L1149-5G, prepared fresh in water), followed by 10 mM DTT to quench excess iodoacetamide. Samples were diluted ∼4 fold (to less than 2 M urea) with 0.1 M Tris pH 8 and digested overnight with 1 mg trypsin to 100 mg protein (Promega V511A, dissolved in 50 mM acetic acid). TFA was added to a final concentration of 0.3–0.1% TFA and the peptides were loaded onto the Sep Pak tC18 column, washed, and eluted with 1 mL 40% ACN/0.1% TFA prior to lyophilization.

For protein abundance analysis samples were resuspended in 90% HILIC buffer B and injected onto a TSKgel amide-80 column (Tosoh Biosciences, 2.0 mm × 15 cm packed with 5 µm particles) equilibrated with 10% HILIC buffer A (2% ACN, 0.1% TFA) and 90% HILIC buffer B (98% ACN, 0.1% TFA) using an AKTA P10 purifier system. The samples were then separated by a one-hour gradient from 90% HILIC buffer B to 55% HILIC buffer B at a flow rate of 0.3 ml/min. Fractions were collected every 1.5 min, combined into 10 fractions, evaporated to dryness, and reconstituted in 0.1% formic acid for mass spectrometry analysis. For phosphorylation analysis, phopshopeptides were further purified using Fe^3+^-IMAC as described previously (Costa *et al.* 2015). Purified phosphopeptides were desalted using C18 STAGE tips, evaporated to dryness, and reconstituted in 0.1% formic acid for mass spectrometry analysis.

Mass spectrometry samples were analyzed in technical duplicate on a Thermo Scientific LTQ Orbitrap Elite mass spectrometry system equipped with a Proxeon Easy nLC 1000 ultra high-pressure liquid chromatography and autosampler system. Raw mass spectrometry data were analyzed using the MaxQuant software package (version 1.3.0.5) (Cox and Mann 2008). Data were matched to SwissProt reviewed entries for *S. cerevisiae* in the UniProt protein database. MaxQuant was configured to generate and search against a reverse sequence database for false discovery rate calculations.

### Western Blot

Standard TCA precipitations were preformed to extract proteins. Samples were resuspended in SDS-PAGE sample buffer, boiled for 10 min, and supernatants were transferred to new tubes. Proteins were subjected to SDS-polyacrylamide gel electrophoresis and transferred to PVDF membranes (Millipore). Western blotting was performed with the following antibodies. Primary antibodies: α-GFP (Clontech #632381); α-Rad53 (Abcam ab104232); α-Flag (Sigma-Aldrich, F3165); α-Myc (BioLegend, #626802); α-Dbf4 (Santa Cruz Biotechnology sc5705); α-eIF2α S51-P (Cell Signaling Technology #97215). All primary antibodies were used at 1:1,000. Secondary antibodies: gαm (BioRad #172-1011), gαr (BioRad #170-6515), dαg (Santa Cruz Biotechnology sc2033) were used at 1:10,000. Blots were visualized by film or LiCor’s Odyssey Imaging System.

### In vitro kinase assay

Cells were collected, washed with water, and resuspended in lysis buffer (0.5% NP-40, 150 mM NaCl, 50 mM Tris-HCl pH 8.0, 5 mM EDTA) supplemented with 0.174 mg/ml PMSF, 5 mM sodium fluoride, 10 mM sodium orthovanadate, 5 mM 2-phosphogylcerol, 1 μg/ml leupeptin, 1 μg/mL bestatin, and 1 μg/mL pepstatin. Cells were lysed with bead beater and separated from beads. Extracts were clarified by centrifugation at 4° (2X). Extracts were quantitated using a BCA protein assay kit (Pierce). Immunoprecipitations were carried out in volumes of 500-600 μL with 0.5 µL of anti-GFP antibody (Abcam, ab190) overnight. Precipitated protein complexes were then recovered with 20 µL Protein A beads (Invitrogen, Dynabeads) for 40 min Beads were washed with lysis buffer with inhibitors 3X followed by two washes with kinase buffer (20 mM Tris-HCl pH 7.5, 20 mM MgCl_2_, 2 mM MnCl_2_, 1 mM DTT, 25 M ATP). Beads are then incubated with γ-^32^P-ATP (Perkin Elmer) plus kinase buffer pre-incubated with 150 nM purified activated Rad53 (30 min at 30°) or no Rad53 for 60 min at 30°. Beads were then washed with lysis buffer 3X to remove kinase, and eluted in 40 μL SDS-PAGE sample buffer with 0.1 M DTT at 65° for 10 min with periodic agitation. Samples were boiled for 5 min prior to loading onto SDS-PAGE gel and transferred to PVDF membrane. Blots were visualized by phosphorimager screen, scanned with a Typhoon phosphoimager (GE Healthcare), and quantified with ImageJ. Westerns were performed to determine protein loading and visualized by LiCor’s Odyssey Imaging System.

### Nuclease assay

Nuclease assay was modified from Pellegrini *et al.* (2008). pRS303, which contains a T7 promoter, was linearized with AlwNI (NEB) and used as a substrate for *in vitro* transcription by PCR using T7 RNA polymerase (NEB) as follows: 5 μL T7 buffer; 22 μL H2O, 2 μL ATP, 2 μL UTP, 2 μL CTP, 0.5 μL GTP, 0.5 μL GTP*, 3 μL GMP, 2 μL RNAsecure (Ambion), 7 μL DNA. Reactions were incubated at 60° for 10 min and cooled to room temperature. 2 μL T7 RNA polymerase was added and reactions were incubated at 37° for 4 hr. After addition of 2 μL DNase, reactions were incubated for another 30 min RNAs were purified with G50 columns twice to remove unincorporated labels, and purified RNA transcripts were added to nuclease reaction buffer (30 mM Tris HCl, pH 8.0, 2 mM MgCl_2_, 50 mM NH_4_Cl, 0.5 mM DTT, 20 μg/mL BSA). Flag purified Xrn1 were added and reactions were allowed to proceed at 37°. Samples were collected after 15, 30, and 60 min Samples were quenched with equal amount of 6X urea loading buffer, incubated at 80° for 10 min, followed by incubation on ice. Samples were loaded onto 10% TBE-UREA gels (Invitrogen; Novex TBE-Urea Gels), and ran at 180V for 35 min The gels were visualized by a Typhoon Phosphoimager (GE Healthcare).

### Northern Blot

RNA was extracted with Qiagen RNeasy kit. Samples were run on 1% agarose gel containing 1.2 M formaldehyde and transferred to Amersham HYBOND+ Nylon membrane overnight, then crosslinked with Stratagene Stratalinker at 254 nm setting. Radioactive probe were made using PCR amplified 5.8S rDNA, Prime It II labeling kit and dCTP* (Perkin-Elmer). Blots were hybridized with Amersham Alkaline Phosphatase Hybridization as indicated, and visualized with a Typhoon phosphoimager.

### S^35^-methionine incorporation

Cells were grown to mid-log phase in C-methionine media and each culture was divided in two. To one half of the cultures, 4-NQO was added to 2 μg/mL for 15 min Subsequently, S^35^-methionine (Perkin Elmers) was added and samples taken at 1 min and 5 min Cells were wash with water, pelleted, and flash frozen on dry ice. Frozen pellets were resuspended in 1X sample buffer and boiled at 100° for 10 min Samples were subjected to SDS-polyacrylamide gel electrophoresis, transferred to PVDF membranes, and visualized by film.

### Ribosome Profiling

Illumina’s ARTseq Ribosome Profiling Kit for yeast (RPYSC12116) was used for ribosome profiling experiments. The size and integrity of the RNA samples were confirmed by Agilent’s Bioanalyzer and submitted to UCSF Center for Advanced Technology for sequencing.

### qPCR

RNA was extracted with Qiagen RNeasy kit and DNase treated with Zymo “DNA-free RNA” kit. cDNA library was generated with BioRad’s iScript RT Supermix for RT-qPCR as instructed and qPCR reaction was performed with BioRad’s SsoFast EvaGreen Supermix as instructed. Primers used: GAL1_FW: TGGTGTTAACAATGGCGGTA; GAL1_RV: GGGCGGTTTCAAACTTGTTA; ACT1_FW: AGGTATCATGGTCGGTATGG; ACT1_RV: ACAAGGACAAAACGGCTTGG; Hst3qPCR_FW: CACAAGTTCATTGCGCAT; Hst3qPCR_RV: CTGCTCCAGGGAAAAGTCTG.

### Spot test

Yeast strains were inoculated into 3-5 ml YPD grown overnight with aeration at 30°. Tenfold dilution series were set up in 96-well plates, and 3 μl aliquots of the dilution series were transferred to plates. Plates were incubated 2-3 days at 30° until colonies formed and then were photographed.

### Data availability

Strains and plasmids are available upon request (Table S1). Supplemental material is available on figshare. Supplemental material of Supplemental data contains a table and four figures. Supplemental Methods contains an additional Materials and Method section. File S1 contains the phosphopeptide dataset comparing *GAL1pr-RAD53 TEF1pr-DUN1* and *rad53∆* cells. File S2 contains the SILAC protein abundance dataset comparing *GAL1pr-RAD53 TEF1pr-DUN1* and *rad53∆* cells. File S3 contains the ribosomal profiling data comparing *rad53∆* cells in HU and *rad53∆* cells untreated. File S4 contains the ribosomal profiling data comparing wild type cells in HU and wild type cells untreated. Supplemental material available at Figshare: https://doi.org/10.25387/g3.7176383.

## Results

### Identification of Rad53 substrates

While previous screens for targets of the DDR kinases, Mec1, Tel1, and Rad53, identified novel targets, a number of known substrates of Rad53, such as Sld3 and Ndd1, were not identified in these screens (Smolka *et al.* 2007; Chen *et al.* 2010; Lopez-Mosqueda *et al.* 2010 p.; Zegerman and Diffley 2010; Edenberg *et al.* 2014; Zhou *et al.* 2016). Therefore, we sought to improve the limit of detection of Rad53 substrates by mass spectrometry by designing a system to saturate the phosphorylation of Rad53 substrates. Most Rad53 substrates that have been characterized have a large number of phosphorylated residues. However, examination of the phosphoproteins by electrophoresis shows a heterogeneous set of shifted bands, suggesting that the sites are not saturated *in vivo*. First, we expressed Rad53 using the inducible galactose (GAL1) promoter to allow for transient overexpression. We found that constitutive overexpression of Rad53 by the *TEF1* or *ADH1* promoters is detrimental to cells, in agreement with published data (Sun *et al.* 1996; Marsolier *et al.* 2000). Additionally, Rad53 in these cells runs at a higher mobility on SDS-PAGE gels indicating a constitutive activation of the checkpoint (data not shown). In addition to Gal1pr-Rad53, we also placed Dun1, a checkpoint kinase that requires Rad53 for activation, under the *TEF1* constitutive promoter, and we deleted *PTC2*, *PTC3*, and *PPH2*, which are phosphatases known to counteract checkpoint phosphorylation (Leroy *et al.* 2003; O’Neill *et al.* 2007; Travesa *et al.* 2008). We expect this to stabilize substrates in the phosphorylated state.

**Figure 1:**
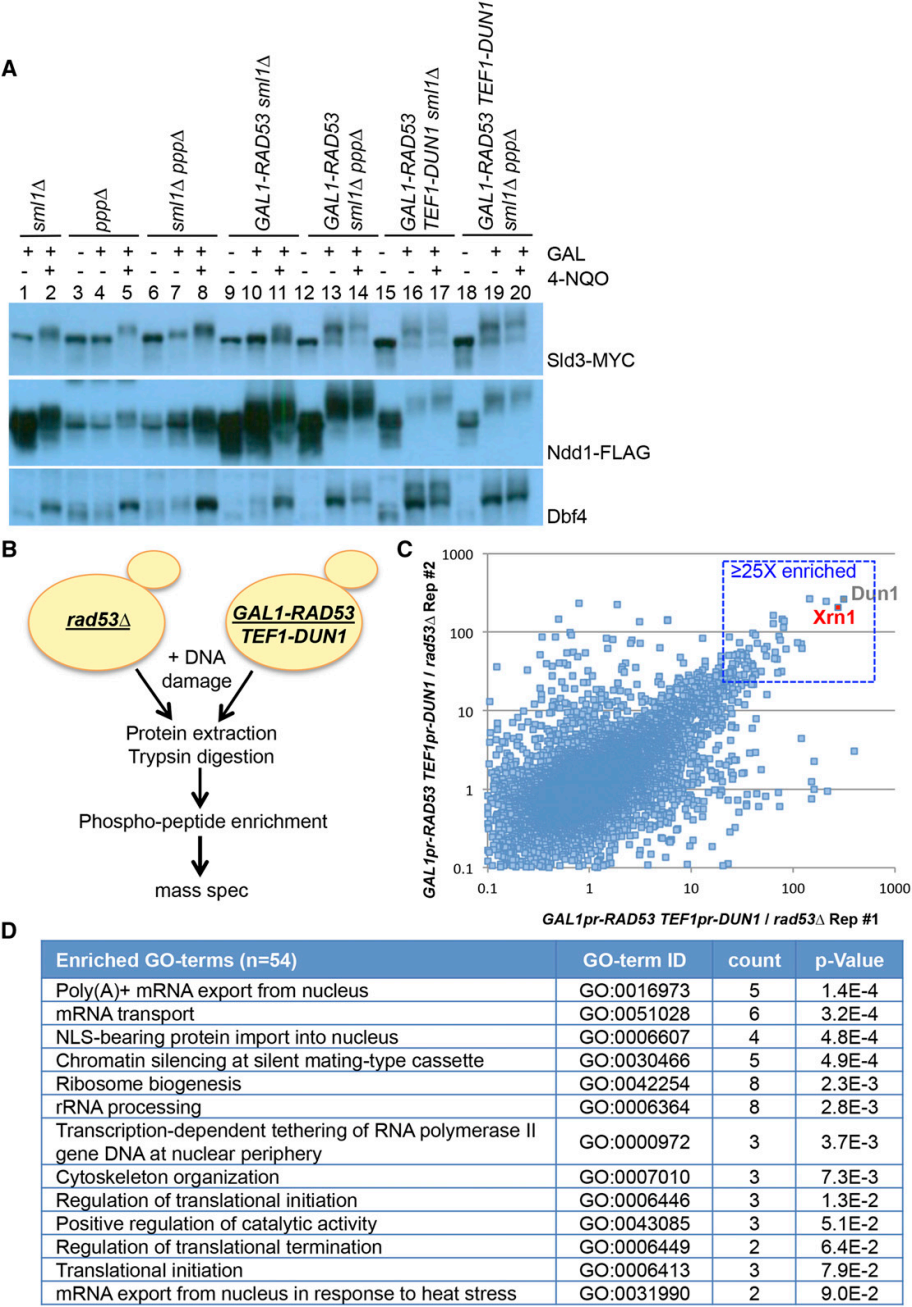
Phosphoproteomic screen for Rad53 targets. A. Western blots showing mobility shift of Rad53 substrates: Sld3, Ndd1, and Dbf4 in the strain indicated. ppp∆ refers to *ptc3∆ ptc2∆ pph3∆*. Sld3 and Ndd1 are respectively tagged with MYC and Flag epitope tags and visualized with antibodies against the tag. Dbf4 is visualized with antibodies against Dbf4. B. Schematic of the experiment for phosphopeptide mass spectrometry. Both strains are in the *sml1∆ ptc3∆ ptc2∆ pph3∆* background. Asynchronous yeast cultures were treated with galactose for 1 hr to induce Rad53 expression followed by the addition of 2 μg/mL 4-NQO for 15 min before harvesting. C. Comparison of fold changes for a phosphopeptide between replicates. Box region shows phosphopeptides that are ≥25 fold enriched in both replicates in *GAL1pr-RAD53 TEF1pr-DUN1* strains. D. Enriched biological process-associated GO terms using DAVID for the top 54 proteins.

Driving Rad53 expression from the *GAL1* promoter leads to phosphorylation of Rad53 substrates Sld3, Ndd1, and Dbf4, even in the absence of DNA damage, indicating that overexpressing Rad53 activates the checkpoint independently of DNA damage (Figure 1A; compare lanes 19 to 20). In contrast, deletion of the phosphatases alone does not lead to phosphorylation of Rad53 substrates in the absence of DNA damage (Figure 1A; lanes 3 and 4). Furthermore, overexpression of Rad53 leads to modification of Rad53 substrates specifically, and not a general DNA damage response. For example, H2AX, a substrate of Mec1, is not significantly phosphorylated when Rad53 is overexpressed (data not shown). In general, Rad53 targets appear much more highly phosphorylated in this system than in wild type cells (compare lane 2 to lane 20 for each substrate). This is consistent with the hypothesis that most Rad53 substrates are only targeted on a subset of their sites upon DNA damage.

For the screen, we used a very short exposure to the DNA damaging agent, 4-nitroquiloine oxide (4-NQO), to avoid accumulation of phosphorylation events downstream of Rad53 due to alteration of cell cycle position (Figure 1B). In contrast, previous proteomics screens for Rad53 substrates treated cells for 2-3 hr in methyl methanesulfonate (MMS) (Smolka *et al.* 2007; Chen *et al.* 2010; Zhou *et al.* 2016). 4-NQO, a UV mimetic drug that distorts DNA by the addition of bulky adducts, rapidly activates the DNA damage checkpoint as detected by Rad53 mobility shift. Cells overexpressing Rad53 and Dun1 (*GAL1pr-RAD53 TEF1pr-DUN1*) and *rad53∆* cells were treated with 4-NQO for 15 min before harvesting. Proteins were extracted, digested with trypsin, and phosphopeptides were enriched using a column based method and identified by mass spectrometry. Two replicates of each experiments were performed. In total, we identified over 29,000 phosphorylated peptides. After filtering out missing values, we quantified more than 13,000 phosphopeptides in all four samples (Figure 1C and Supplemental File S1). In addition to the expected Dun1 phosphopeptides, an Xrn1 exoribonuclease phosphopeptide was highly enriched (>200 fold) in both replicates of the experiment (Figure 1C).

**Figure 2:**
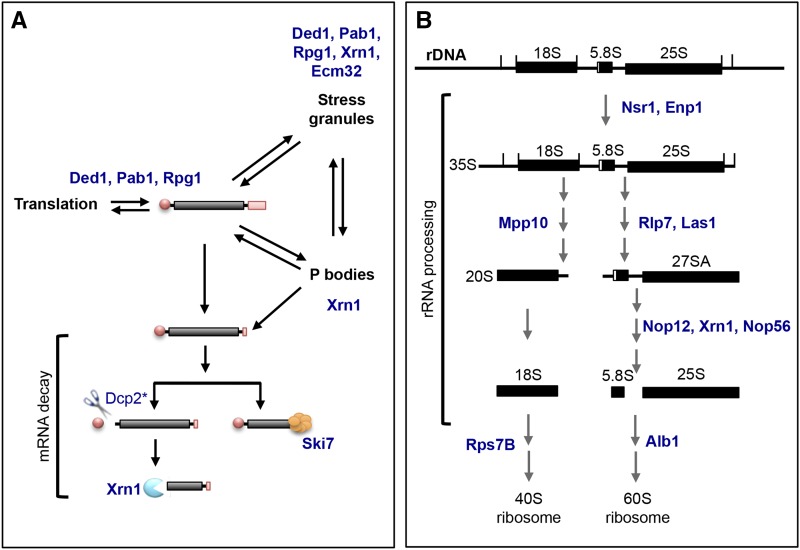
Schematic of RNA processing pathways. A. Schematic of mRNA dynamics in the cell. mRNA is dynamically regulated in the cell, and can be translated into proteins, stored in stress granules, degraded in the processing bodies (P-bodies), or undergo mRNA decay outside of the P-bodies. Proteins whose phosphopeptides were ≥25 fold enriched in our phosphoproteomics were placed in the processes in which they are known to be involved (in blue). *Dcp2 is 20X enriched in *GAL1pr-RAD53 vs. rad53∆*. B. Schematic of rRNA processing in the cell. The 35S pre-rRNA transcript undergo a series of distinct processing events to generate the rRNA components of the 40S ribosome (18S) and 60S ribosome (5.8S and 25S). The 5S component of the 60S ribosome is independently transcribed and not shown. Proteins whose phosphopeptides were ≥25 fold enriched in our phosphoproteomics are indicated (in blue).

To control for changes in protein abundance in response to DNA damage, we carried out a SILAC (stable isotope labeling with amino acids in cell culture) experiment concurrently. The majority of proteins did not change significantly between *GAL1pr-RAD53* and *rad53∆* cells (Supplemental File S2). Our dataset includes an enrichment of proteins that are involved in RNA biology or early translation events, reinforcing the idea that these pathways are regulated by the DDR in damage (Figure 1D, Figure 2). For example, five of the 54 substrates, Ded1, Pab1, Xrn1, Rpg1, and Ecm32 are mRNA binding proteins and components of stress granules, while Ded1, Pab1, and Rpg1 are also important for mRNA stability and translation (Figure 2A) (Kedersha *et al.* 2005; Decker and Parker 2012; Mitchell *et al.* 2012). Dcp2, an mRNA decapping enzyme that narrowly missed our 25-fold enrichment cutoff, is required for the removal of the 5′ cap on the mRNA in order to generate the substrate for Xrn1 (Braun *et al.* 2012). On the other hand, Ski7 is a component of the exosome complex that also degrades mRNA (van Hoof *et al.* 2000; Araki *et al.* 2001). Similarly, 10 of the 54 substrates Nsr1, Enp1, Mpp10, Rlp7, Las1, Nop12, Xrn1, Nop56, Rps7B, and Alb1 are involved in 35S rRNA processing and ribosome biogenesis (Figure 2B) (Schillewaert *et al.* 2012; Woolford and Baserga 2013; Gasse *et al.* 2015). The enrichment of substrates in these pathways suggests that they are *bona fide* Rad53 substrates.

### The DNA damage checkpoint does not affect rRNA processing or translation

**Figure 3:**
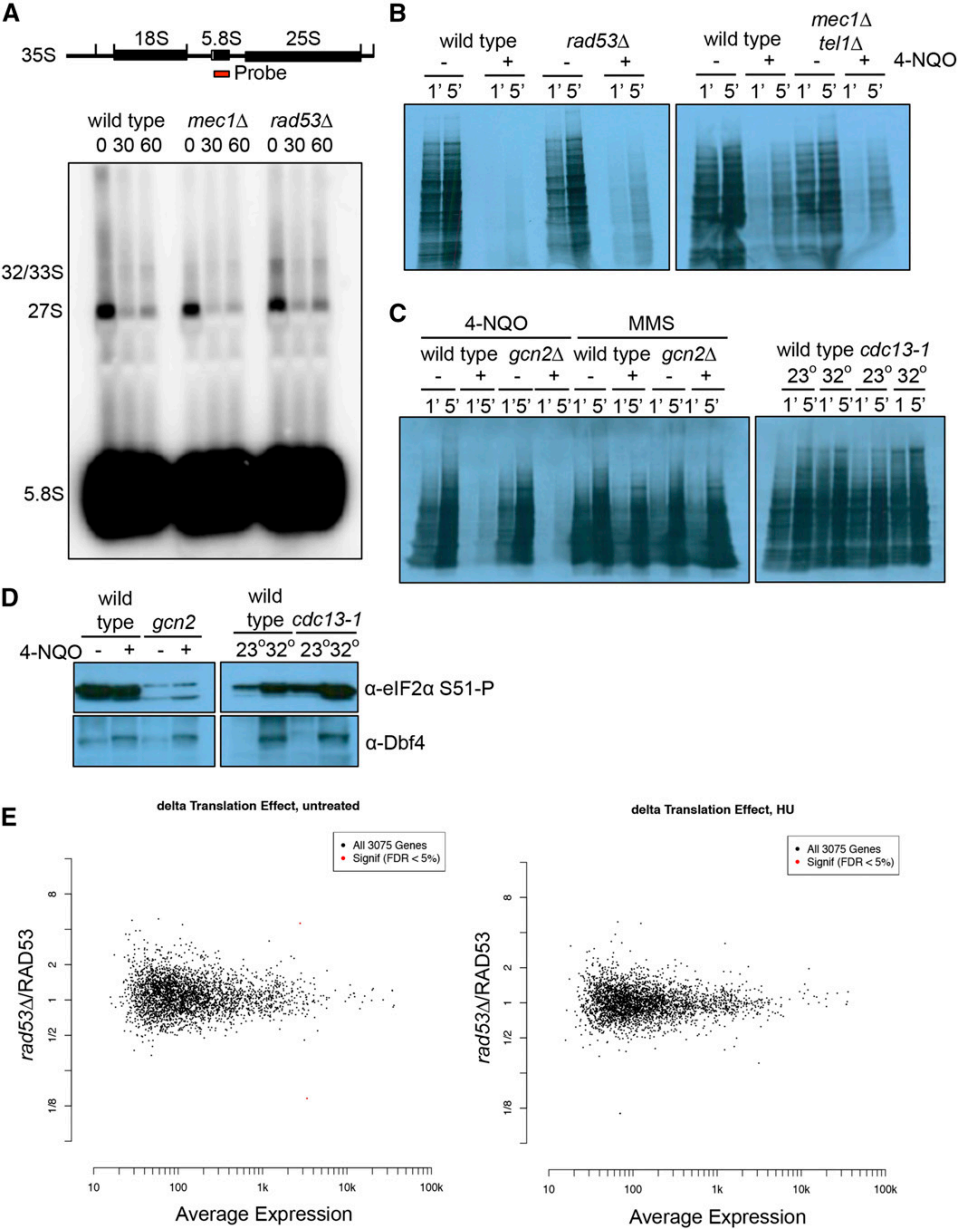
Downregulation of rRNA processing or protein synthesis is independent of the DNA damage checkpoint. A. Wild type, *mec1∆*, and *rad53∆* cells are untreated or treated with 2 μg/mL 4-NQO. Samples were collected at 30 min and 60 min after treatment for RNA extraction. The full length 35S pre-rRNA transcript is processed to yield mature 18S, 5.8S, and 25S rRNA. Northern blot with probe against full-length 5.8S sequence allows detection of the 32/33S and 27S intermediates, and the fully processed 5.8S rRNA. B. Film of S^35^-methionine incorporation. Wild type, *rad53∆*, and *mec1∆ tel1∆* cells were untreated or treated with 2 μg/ml 4-NQO for 15 min S^35^-methionine was added and samples were taken after 1 min and 5 min. C. Film of S^35^-methionine incorporation. Left panel: wild type and *gcn2∆* cells were untreated or treated with 2 μg/ml 4-NQO for 60 min Middle panel: G1 arrested wild type and *gcn2∆* cells were untreated or treated with 0.05% MMS for 60 min Right panel: G1 arrested wild type and *cdc13-1* cells were grown at 23°C or 32°C for 2 hr, the permissive and nonpermissive temperature for *cdc13-1* respectively. S^35^-methionine was added and samples were taken after 1 min and 5 min. D. Western blot of eIF2 alpha phosphorylation. Left planel: wild type and *gcn2∆* cells were untreated or treated with 2 μg/ml 4-NQO for 60 min Right panel: G1 arrested wild type and *cdc13-1* cells were grown at 23°C or 32°C for 2 hr. Samples were blotted for phosphorylated eIF2 alpha-S51 and Dbf4. E. Wild type and *rad53∆* cells were arrested in G1 with α factor and released into rich media or rich media with 200 mM HU for 45 min before harvesting. Total RNA and ribosome footprints were purified using Illumina’s ARTseq kit before sequencing. Left panel is an M-A plot showing the average expression for a gene between *rad53∆* and wild type cells (x-axis) *vs.* the fold change between *rad53∆* and wild type cells (y-axis) in the absence of DNA damage. Right panel is an M-A plot showing the average expression for a gene between *rad53∆* and wild type cells (x-axis) *vs.* the fold change between *rad53∆* and wild type cells (y-axis) in the presence of HU.

The enrichment of proteins involved in cytoplasmic RNA maintenance and degradation suggests that Rad53 regulates RNA metabolism through many distinct targets to ensure a robust mechanism of regulation (Figure 1D). If this is true, we reasoned that there might be a direct effect on RNA levels, translation efficiency, or ribosome biogenesis. To determine the effect of DNA damage on rRNA processing, Northern blots of full-length 35S and 27S pre-rRNA transcripts were visualized by probing with the 5.8S sequence. Treatment with 4-NQO caused a strong reduction in the amount of unprocessed rRNA in a *MEC1* and *RAD53* independent manner (Figure 3A). This is presumably due to a loss of rDNA transcription coupled with processing, but this loss did not depend upon *RAD53* or *MEC1* and *TEL1* (Figure 3A). Next, we looked at global translation by S^35^-methionine incorporation of newly synthesized transcripts. Protein synthesis is inhibited by UV irradiation in fission yeast, and this has been reported to require Gcn2 (Tvegård *et al.* 2007). We found that protein synthesis in budding yeast was strongly inhibited by 4-NQO, but not MMS (Figure 3B and C). However, this was unaffected by deletion of *GCN2*, *RAD53*, or *MEC1* and *TEL1*, suggesting it might represent a physical block to the translation machinery due to mRNA damage caused by 4-NQO (Figure 3C and D). To control for any potential damage to mRNA caused by chemical agents, we use the *cdc13-1* mutation, which, at the non-permissive temperature, causes the accumulation of single-stranded DNA near the telomeres that activates the DNA damage checkpoint (Garvik *et al.* 1995; Lydall and Weinert 1995). This mutation caused very little effect on methionine incorporation, although the heat shock required to inactivate the *cdc13-1* allele induced some eIF2 alpha phosphorylation. However, the presence of the *cdc13-1* allele did not further increase the level of phosphorylated eIF2 alpha (Figure 3E).

As we saw no effect of the DDR on the global translation rate, we performed ribosome profiling to determine whether specific transcripts, or classes of transcripts, were being regulated by Rad53. In humans, DNA damage is thought to reprogram translation to allow the selective translation of a subset of messages (Jousse *et al.* 2003; Holcik and Sonenberg 2005; Kruiswijk *et al.* 2012). We used ribosome profiling to examine ribosome occupancy in *RAD53 vs. rad53∆* cells to determine whether the DNA damage checkpoint pathway affected translation directly. To avoid any possible negative effect of 4-NQO on mRNA structure, we used hydroxyurea (HU) to deplete the dNTP pool, which causes accumulation of ssDNA and activation of the DDR (Santocanale and Diffley 1998). Cells were arrested in G1 and released in HU for 45 min, and both total mRNA and ribosome-protected footprints were sequenced. We examined the translation rate for each message by normalizing ribosome-bound transcript to total mRNA for each gene. Figure 3E compares this translation rate for each transcript between *rad53∆ vs.* wild type (*y*-axis) as compared to the mean transcript level (*x*-axis). We found no significant evidence for translational regulation by Rad53 after normalization of *RAD53*-dependent transcriptional effects in either the presence or absence of HU (Figure 3E; Supplemental File S3 and S4). Thus, although there is an enrichment of proteins involved in cytoplasmic RNA maintenance and degradation in our list of Rad53-dependent phosphopeptides, Rad53 does not seem to be generally required for normal rRNA processing or translation.

### Xrn1 is an in vitro and in vivo substrate of Rad53

**Figure 4:**
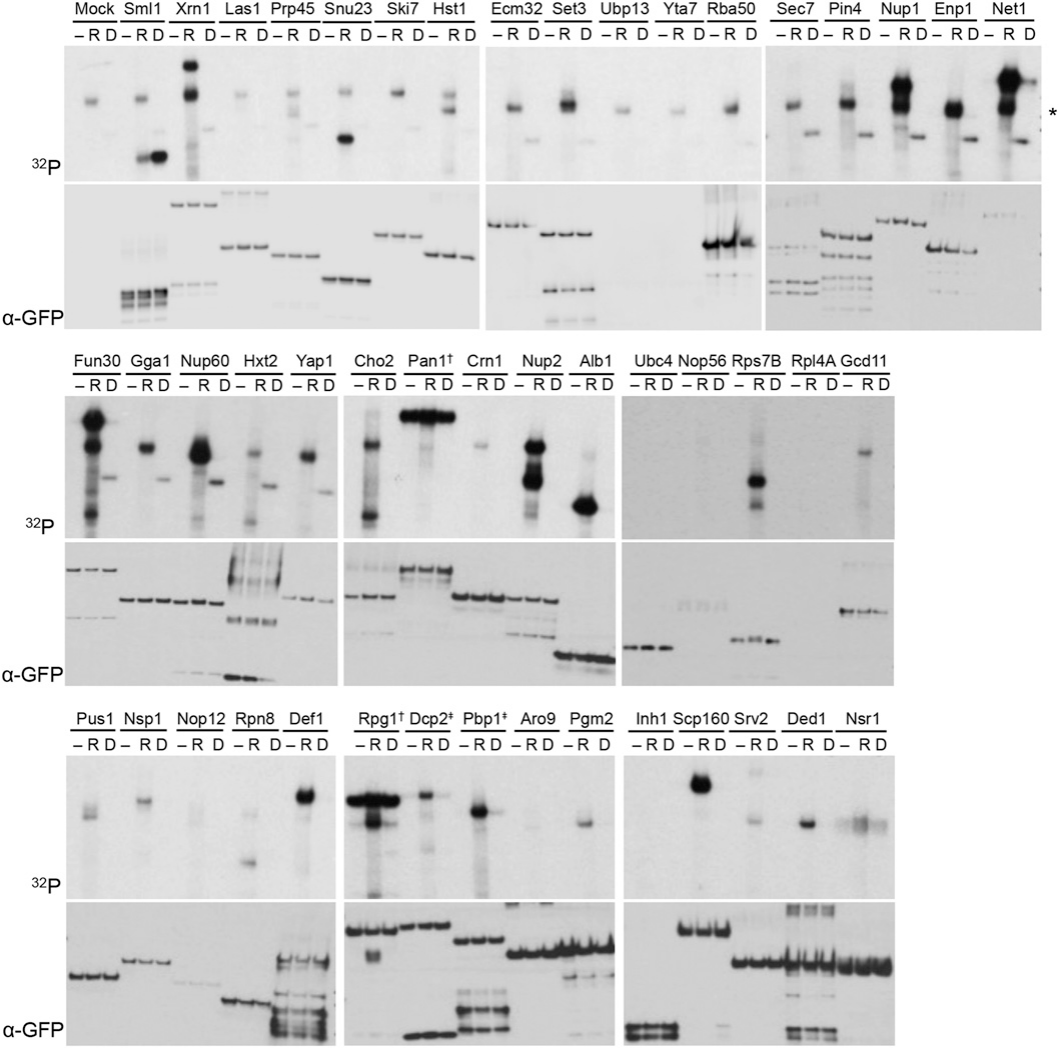
Validation by *in vitro* kinase assay of proteins associated with enriched phosphopeptides in the screen. A. *In vitro* kinase assay for 44 proteins whose phosphopeptides were enriched by ≥25 fold and where the GFP-tagged strains were available in the GFP collection. For each protein set, - indicates no kinase control, R indicates Rad53, and D indicates Dun1. Top panels are autorads showing ^32^P signal indicating transfer of ^32^P-ATP to substrate. * indicates Rad53 autophosphorylation signal. ^†^ indicates contaminating kinase in IP. Bottom panels show Western blot for GFP as loading control for each lane. ^‡^ Dcp2 (20X enriched) and Pbp1 (2X enriched) were selected because of their connection to Xrn1 in P body biology.

We identified 54 proteins corresponding to phosphopeptides that were at least 25-fold enriched in *GAL1pr-RAD53* compared to *rad53∆* cells (Table 1). A 25-fold enrichment cutoff was selected arbitrarily for a workable set of protein candidates to follow up in our *in vitro* kinase assay. 21 of these 54 proteins are previously known or identified substrates of Rad53 and 33 of these are novel substrates as indicated in bold (Table 1). The observation that some of the identified phosphopeptides are within previously known Rad53 substrates validates our screening technique. For example, the nucleoporins, Nup1, Nup2, and Nup60 are frequently observed in screens for Rad53 and DDR targets, which points to a role of the DDR in regulating mRNA nuclear export, as suggested by Zhou *et al.* 2016 (Smolka *et al.* 2007; Chen *et al.* 2010; Zhou *et al.* 2016). Pin4, on the other hand, is a known DDR target that is phosphorylated by Mec1 and Tel1 (Pike *et al.* 2004). Zhou *et al.*, (2016) identified Pin4 phosphopeptides enriched in wild type *vs. rad53∆* cells treated with MMS. Our data also suggest that Pin4 is also a direct substrate of Rad53 (Table 1 and Figure 4). Ded1, on the other hand, was only twofold enriched (L/H ratio of 0.4969) in the published *rad53∆/*wild type dataset and fell below the fourfold enrichment cutoff that was set (Zhou *et al.* 2016). Comparing our dataset to those of Smolka *et al.*, (2007), Chen *et al.*, (2010), and Zhou *et al.*, (2016) as shown in Table 1, there are few proteins identified in three or more of the four datasets. This suggests that each dataset is subject to its own caveats, reinforcing the value of conducting this screen multiple times in subtly different ways. Furthermore, given that our screen was done with 4-NQO, as opposed to MMS, and our cells were subjected to only fifteen minutes of DNA damage *vs.* two to three hours, we would expect some differences between the datasets. For example, our analysis may miss sites that accumulate over time, but will also avoid potential indirect effects associated with *RAD53*-dependent cell cycle arrest after long time points. To this end, we followed up with *in vitro* kinase assays to determine which substrates were direct targets of Rad53 or Dun1 (described below).

**Table 1:** Unique phosphopeptides that are enriched ≥25 fold in *GAL1-RAD53/rad53∆*.

**Protein**	**Phospho-site**	**OX/∆ 1**	**OX/∆ 2**	**SILAC OX/∆**	**IVK**	**References**
**Xrn1**	S1467	277	206	1	+	This study
Pin4	S189, S190, S191	146	263	1	-	This study; Zhou et al., 2016 (Asynchronous)
**Mpp10**	S299	79	144	1	ND	This study
Nup2	S316	82	138	1	++	This study; Smolka et al, 2007; Chen et al., 2010
**Pab1**	S565	64	161	1	ND	This study
Npl3	S224	81	114	1	ND	This study; Smolka et al, 2007
Pgm2	S2	64	127	2	+	This study; Smolka et al, 2007
**Gcd11**	S258	122	62	1	+	This study
Npl3	S224	73	88	1	ND	This study; Smolka et al, 2007
Fun30	S98	75	79	1	++	This study; Chen et al., 2010
Nsr1	S405, S409	86	60	1	ND	This study; Zhou et al., 2016
**Rps7B**	S168	40	97	1	++	This study
Net1	T838	43	91	1	++	This study; Smolka et al., 2007; Zhou et al., 2016
**Rlp7**	S14	55	65	1	ND	This study
**Gga1**	S185	45	72	1	-	This study
Def1	S273	53	61	1	++	This study; Smolka et al, 2007
**Nop56**	S317	70	45	1	-	This study
**Sec7**	S215, S218	66	46	1	-	This study
Nsp1	S285	55	49	1	+	This study; Smolka et al., 2007; Zhou et al., 2016
**Ded1**	S218	77	32	ND	+	This study
**Cho2**	S598	30	73	1	+	This study
Nsp1	S532	70	30	1	+	This study; Smolka et al., 2007; Zhou et al., 2016
Hxt2	S11, S13	24	86	1	-	This study; Zhou et al., 2016 (S-phase)
Nsp1	S323	34	53	1	+	This study; Smolka et al., 2007; Zhou et al., 2016
Rlp7	S278	31	55	1	ND	This study; Zhou et al., 2016 (S-phase)
Nsp1	S551	35	47	1	+	This study; Smolka et al., 2007; Zhou et al., 2016
Ski7	S88, S90	32	51	ND	-	This study; Zhou et al., 2016
**Ubc4**	S12	31	50	ND	-	This study
**Pan1**	S745	39	39	1	ND	This study
**Rpn8**	S314	49	31	1	-	This study
**Prp45**	S370	40	37	ND	-	This study
Net1	S747	36	42	1	++	This study; Smolka et al., 2007; Zhou et al., 2016
YLR257W	S137, S139	40	37	1	ND	This study; Zhou et al., 2016 (Asynchronous 1X)
Nup60	S63	28	52	1	++	This study; Smolka et al., 2007; Zhou et al., 2016
**Rpg1**	S872	36	37	1	ND	This study
Nsr1	S405	26	50	1	ND	This study; Zhou et al., 2016
**Rrd1**	S385	34	38	ND	ND	This study
Hsp42	S213, S214	401	3	1	ND	This study; Zhou et al., 2016
Nup1	S754	43	29	1	++	This study; Smolka et al, 2007; Chen et al., 2010
**Inh1**	S38	28	43	1	-	This study
Nup2	S68	35	34	1	++	This study; Zhou et al., 2016 (Asynchronous 1X)
Enp1	S404	23	50	1	++	This study; Chen et al., 2010
**Pus1**	S478	13	83	1	+	This study
Net1	S785	27	39	1	++	This study; Smolka et al, 2007; Chen et al., 2010
**Hst1**	S143	40	26	ND	+	This study
**Tub2**	S280	40	25	1	ND	This study
**Crn1**	S462	25	39	1	-	This study
Yta7	S1290	20	49	1	ND	This study; Smolka et al., 2005 and Smolka et al., 2006 showed Yta7 binds Rad53 in MMS
Gle1	S108	33	29	ND	ND	This study; Zhou et al., 2016
**Aro9**	S502	55	16	ND	-	This study
**Nop12**	S2	41	22	1	-	This study
Ecm32	S206	25	34	ND	-	This study; Zhou et al., 2016
**Ubp13**	S198	39	22	ND	ND	This study
**Srv2**	S346	6	142	1	+	This study
**Set3**	S741	30	28	ND	+	This study
**Snu23**		26	32	ND	+	This study
**Srv2**	S343	20	41	1	+	This study
**Alb1**	S41	26	31	ND		This study
**Rba50**	S233	34	23	1		This study
**Las1**	S467	24	30	ND		This study
Yap1	S503	24	30	1	-	This study; Zhou et al., 2016 (Asynchronous 1X)
Rrp4A	T60	31	22	1	ND	This study; Zhou et al., 2016 (S-phase)
Ecm32	S206	27	25	ND	-	This study; Zhou et al., 2016 (S-phase)
Nsp1	S221	31	21	1	+	This study; Zhou et al., 2016 (S-phase)
**Sec53**	S54	18	35	1	ND	This study
**Scp160**	S325	21	29	1	++	This study

In 8/54 cases, multiple phosphosites were identified for the same substrate. The enrichment of these peptides is not due to increased protein abundance in *GAL1pr-RAD53* cells, as indicated by the SILAC ratio between *GAL1pr-RAD53* and *rad53∆*. In order to determine whether these were direct substrates of Rad53 or Dun1, we examined their phosphorylation by these kinases *in vitro*. Activated epitope tagged Rad53 and Dun1 were purified from cells treated with 4-NQO. As a control for Dun1 phosphorylation, we used the Dun1 substrate Sml1. Sml1 was strongly phosphorylated by Dun1 and poorly phosphorylated by Rad53 *in vitro* (Figure 4; top left panel). We purified 44 substrates that were available in the GFP collection and found that 21 were moderate to strong Rad53 substrates *in vitro*. No substrate tested was phosphorylated by Dun1, suggesting that this kinase may have a much more limited set of targets (Figure 4). Specifically, Xrn1 is a strong *in vitro* substrate of Rad53. Xrn1 was previously found in the set of over 13,000 peptides identified in a SILAC screen comparing phosphopeptides between *rad53∆* cells and wild type cells treated with MMS (Zhou *et al.* 2016). However, Xrn1, along with a very large number of other proteins, was not within their fourfold enrichment cutoff and was therefore not considered significant. In our phosphoproteomics screen, Xrn1 is over 200-fold enriched in *GAL1pr-RAD53 vs. rad53∆* cells.

**Figure 5:**
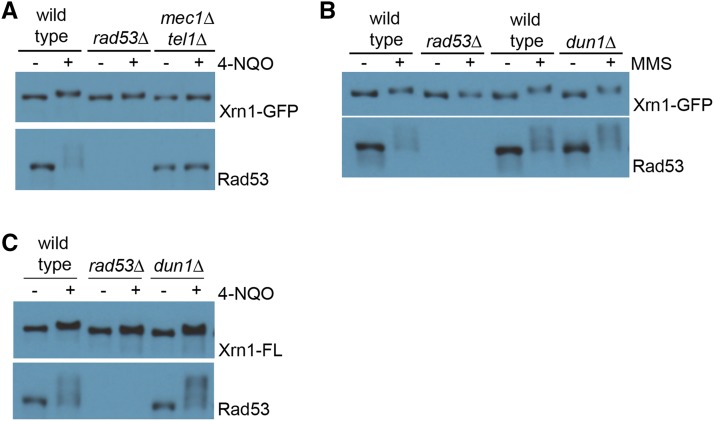
Rad53 is required for Xrn1 mobility shift during DNA damage. A. Western blot showing Xrn1-GFP shift upon DNA damage, and is dependent on Rad53 and Mec1/Tel1. Asynchronous yeast cultures were treated with 2 μg/mL 4-NQO for 90 min before harvesting. B. Western blot showing Xrn1-GFP shift is independent of Dun1. Asynchronous cells were treated with 0.05% MMS for 3 hr before harvesting. C. Western blot showing Xrn1-Flag shift is dependent on Rad53, but independent of Dun1. Asynchronous cells were treated with 2 μg/mL 4-NQO for 90 min. Rad53 is shown as a control for DNA damage treatment, because it hyper-shifts in response to DNA damage.

Because we identified Xrn1 in a screen in which Rad53 was over-expressed, we wished to confirm Xrn1 phosphorylation *in vivo*. We epitope tagged Xrn1 on its C terminus with either GFP or Flag and examined its electrophoretic mobility after treatment with damaging agents. Phosphorylation of Xrn1 is DNA damage-dependent *in vivo*, as detected by a mobility shift. Consistent with Xrn1 being a direct substrate of Rad53, its modification in DNA damage is dependent on Rad53 and the upstream sensor kinases, Mec1 and Tel1 (Figure 5A, B). In contrast, Dun1 is not required for Xrn1 mobility shift in DNA damage (Figure 5B, C). This modification is independent of DNA damage type as Xrn1 shifts in both 4-NQO and MMS. Given the strength of the Xrn1 signal *in vivo* and *in vitro*, we characterized this target further.

### Phosphorylation of Xrn1 does not noticeably alter its nuclease activity

**Figure 6:**
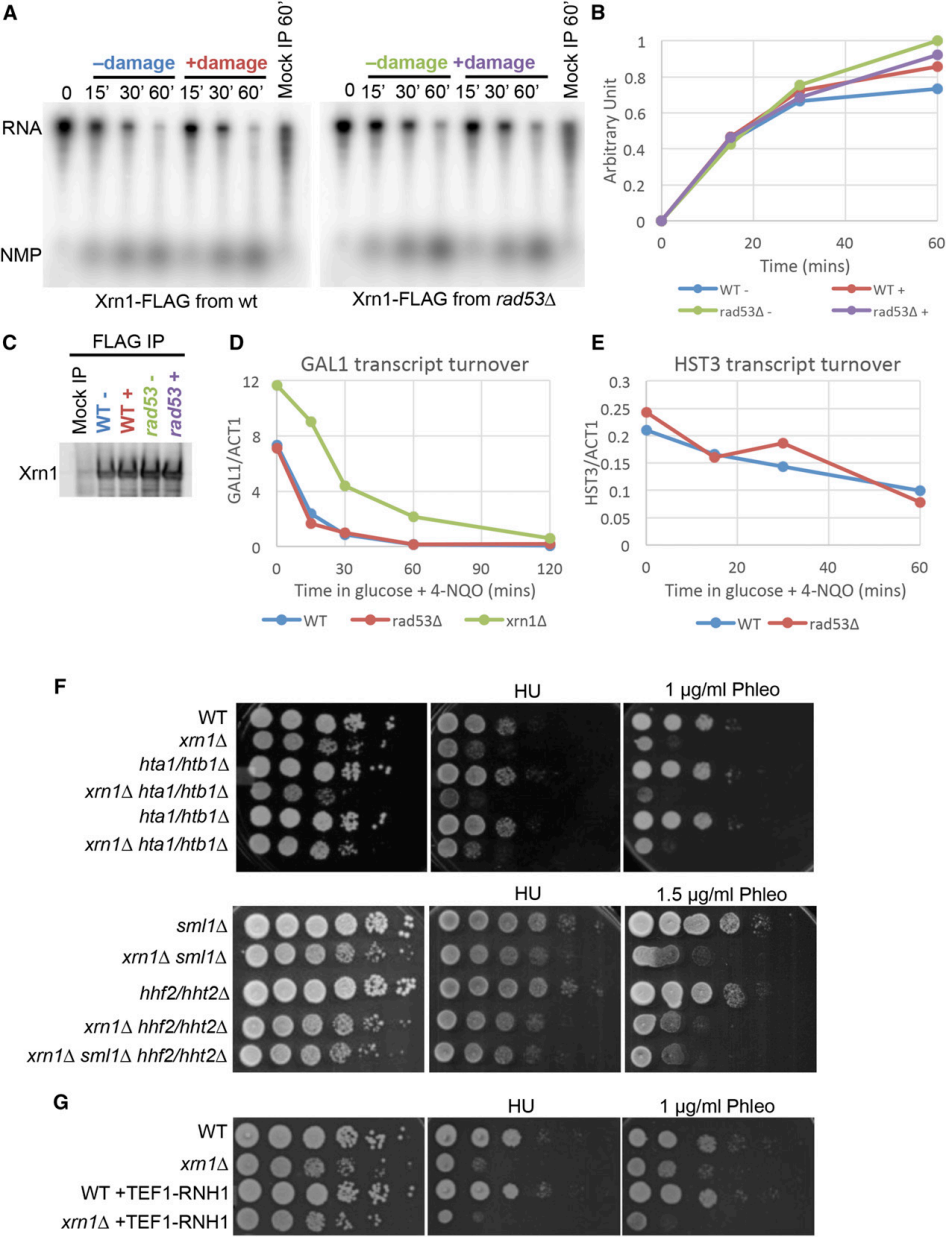
Xrn1 phosphorylation does not interfere with its nuclease activity *in vitro*, or its ability to degrade endogenous transcripts. A. Nuclease assay using *in vitro* transcribed RNA as substrate with purified Xrn1 from cells untreated (-) and treated (+) with 2 μg/mL 4-NQO for 1 hr. Nuclease assay was allowed to proceed for 60 min and samples were collected at the indicated time. Mock IP sample was allowed to proceed for 60 min B. Quantification of product (NMP) formation normalized to t = 0 signal. C. Western blot showing relative Xrn1 abundance used in experiment in A. D. Normalized mRNA levels of *GAL1* transcript turnover. The *GAL1* promoter is rapidly repressed when galactose in the media is replaced with glucose. 4-NQO was added to a concentration of 2 μg/mL concurrently with glucose and samples were collected at the indicated time. E. Normalized mRNA levels of *HST3* transcript turnover. *HST3* is placed under the *GAL1* promoter, which is downregulated in glucose and the rate of degradation of *HST3* was determined. 4-NQO was added to a concentration of 2 μg/mL concurrently with glucose and samples were collected at the indicated time. F. Tenfold serial dilutions of the indicated strains grown on YPD, YPD + 100 mM HU, or YPD + 1 μg/mL phleomycin (or YPD + 1.5 μg/mL phleomycin). Plates were grown at 30°C for 2-3 days before scanning. G. Tenfold serial dilutions of the indicated strains grown on YPD, YPD + 100 mM HU, or YPD + 1 μg/mL phleomycin. Plates were grown at 30°C for 2-3 days before scanning.

Next, we sought to determine how this modification of Xrn1 affects Xrn1 function. Post-translational modification of a protein can alter its biochemical activity, localization, or stability. To examine whether phosphorylation altered Xrn1’s nuclease activity, we purified Xrn1 from untreated cells or cells treated with 4-NQO and examined its activity in an *in vitro* nuclease assay. We found that Xrn1 purified from untreated wild type cells, cells treated with 4-NQO, and *rad53∆* cells had similar nuclease activity *in vitro* (Figure 6A, B, C). Similarly, phosphorylation of Xrn1 *in vitro* by Rad53 had no effect on Xrn1’s nuclease activity (data not shown). Thus, we are not able to determine the effect of Rad53 phosphorylation on the *in vitr*o nuclease activity of Xrn1.

We wondered whether the effect of Rad53 phosphorylation on Xrn1 might not be observable in an *in vitro* system. To overcome the possibility that purification of Xrn1 may perturb its regulatory mechanism, we examined turnover of transcripts *in vivo*. First, we followed mRNA degradation of the *GAL1* transcript in 4-NQO-treated cells. *GAL1* transcript is strongly expressed when cells are grown in galactose, but is quickly repressed when glucose is available. *GAL1* mRNA had both a higher steady state level and a longer half-life in *xrn1∆* cells (33 min) compared to wild type cells (17.1 min) (Figure 6D). However, *RAD53* deletion did not strongly affect the mRNA half-life (15.5 min) (Figure 6D). We also examined mRNA turnover for *HST3* message. The *HST3* gene is regulated by the DDR transcriptionally and post-translationally, and thus it was a reasonable candidate for a message that would be targeted by the DDR (Edenberg *et al.* 2014). We removed the ability of the DDR to regulate *HST3* transcription by replacing the *HST3* promoter with the *GAL1* promoter. Upon shifting the cells to glucose media to inactivate the GAL1 promoter, we found that the turnover kinetics of the *HST3* transcript were similar in wild type (50.8 min) and *rad53∆* cells (44 min) exposed to 4-NQO (Figure 6E). Next, we tested another mRNA candidate that might be targeted by Xrn1. Histone gene depletions have been shown to rescue *rad53∆* and *lsm1∆* mutants (Gunjan and Verreault 2003; Herrero and Moreno 2011). We wondered if this is mediated by Xrn1. We found that histone gene deletion did not rescue the slow growth of *xrn1∆* cells or their DNA damage sensitivity (Figure 6F), suggesting that histone genes are not the critical target of Xrn1.

Xrn1 is required for normal levels of RNA in the cell and *xrn1∆* cells have significantly higher levels of RNA, especially small cryptic RNA species (van Dijk *et al.* 2011; Sun *et al.* 2013). We wondered whether excess RNAs might promote formation of RNA:DNA hybrids. If Xrn1 loss promoted the accumulation of RNA:DNA hybrids, overexpressing RNase H (Rnh1), which specifically degrades RNA:DNA hybrids, should rescue the DNA damage sensitivity of *xrn1∆* cells. To examine this, we integrated an additional copy of Rnh1, driven by the TEF1 promoter. However, this did not rescue the sensitivity of *xrn1∆* cell to phleomycin (Figure 6G), indicating that it is unlikely that the DNA damage sensitivity of *xrn1∆* is due to high levels of RNA:DNA hybrids.

**Figure 7:**
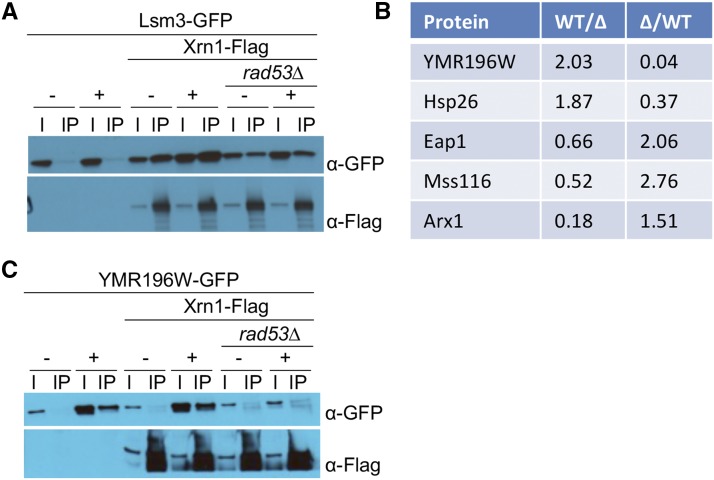
Change in Xrn1 interactions in *rad53∆* cells. A. Co-IP of Xrn1-Flag and Lsm3-GFP in wild type or *rad53∆* cells. Asynchronous cells were untreated (-) or treated (+) with 0.05% MMS for 3 hr. Samples were immunoprecipitated with Flag antibodies. B. List of proteins identified in reciprocal SILAC mass spectrometry analyses of Xrn1-Flag IP from wild type and *rad53∆* cells treated with 2 μg/mL 4-NQO for 60 min. C. Co-IP of Xrn1-Flag and Mss116-GFP or YMR196W-GFP in wild type or *rad53∆* cells, as indicated. Asynchronous cells were untreated (-) or treated (+) with 0.05% MMS for 3 hr, and samples were immunoprecipitated with Flag antibodies.

Since Xrn1 is a component of the P-bodies, we asked whether the phosphorylation of Xrn1 affects its ability to be incorporated into P-bodies during DNA damage (Sheth and Parker 2006; Parker and Sheth 2007; Teixeira and Parker 2007; Buchan *et al.* 2008; Franks and Lykke-Andersen 2008). We treated wild type and *rad53∆* cells with 4-NQO to determine whether Rad53 affects the localization of Xrn1-GFP upon DNA damage. We found that Xrn1 forms distinct GFP foci during DNA damage, and that this is independent of Rad53 (Figure S1). To determine whether DNA damage affects the interaction of Xrn1 with P-body components, we performed co-IPs of Flag-tagged Xrn1 with a number of P-body proteins that are available in the yeast GFP collection in the absence or presence of MMS. Interaction of Xrn1 with Pat1, Edc3, Scd6, Nrp1, Pbp1, Rpg1, YGR250C/Rie1, Npl3, and Ngr1 do not change during DNA damage or in the absence of Rad53 (Figure S2). However, the level of YGR250C/Rie1 increased in DNA damage, as previously described (Tkach *et al.* 2012). Of note, we found that in the absence of Rad53, the interaction between Xrn1 and Lsm3 is reduced, but this does not appear to depend on DNA damage (Figure 7A).

Because a candidate based approach to determine the effect of Rad53 phosphorylation on Xrn1 proved inconclusive, we sought to identify changes in interaction partners using SILAC IP followed by mass spectrometry. After treatment with 4-NQO to induce DNA damage, we purified Xrn1-Flag from wild type cells grown in light labeled arginine and lysine and *rad53∆* cells grown in heavy labeled arginine and lysine. A replicate was done in the reciprocal direction, where wild type cells were grown in heavy labeled arginine and lysine and *rad53∆* cells were grown in light labeled arginine and lysine. We found five proteins that show a reciprocal change in Xrn1 association: YMR196W, Hsp26, Eap1, Mss116, and Arx1 (Figure 7B). We examined each of these interactions by co-immunoprecipitation of GFP-tagged alleles of candidates with Xrn1-Flag. We could not detect a DNA damage-dependent change in the interaction of Hsp26, Eap1, or Arx1 with Xrn1 (Figure S3). Interestingly, similar to Lsm3, interaction of Xrn1 with Mss116 is altered in *rad53∆* independently of DNA damage (Figure 7C). In contrast, expression of YMR196W appears to be strongly induced by DNA damage in a *RAD53*-dependent manner (Figure 7C).

To understand the role of Xrn1 during DNA damage, we sought to identify the residues that are phosphorylated by Rad53. Our original screen identified a single site on Xrn1 (S1467) that was enriched in *GAL1pr-RAD53* cells (Table 1). Deletion of a small portion of the C terminus (1396-1528) that includes this residue was not sufficient to abolish the Xrn1 shift in DNA damage, nor did it result in any damage sensitivity (Figure S4A and S4B). To further characterize the phosphorylation sites, we phosphorylated purified Xrn1 with Rad53 *in vitro* and mapped the *in vitro* sites by mass spectrometry. Six sites in the C-terminal region corresponding to amino acids 1155 – 1330 showed strong enrichments (S1155, T1268, S1270, S1306, S1328, S1329). However, within this 175 amino acid region, there are 32 serine and threonine residues. Since Rad53 does not have a strong consensus sequence and our coverage in this region of the protein was poor, we mutated all 32 residues to alanine (32A). Additionally, we truncated the C terminus (1331-1528), which contains phosphorylation site S1467 identified in our original screen in combination with the 32A mutations (32A-∆C). Both of these mutants are sensitive to DNA damage (Figure S4B). The 32A-∆C cells are as sensitive to phleomycin as the *xrn1∆* cells, while the 32A mutant shows an intermediate phenotype. However, these mutants were slow-growing even in the absence of phleomycin, suggesting that these Xrn1 mutants may not be functioning normally. Therefore, we performed an *in vitro* assay and determined that the nuclease activity itself is defective in these mutants (Figure S4C and S4D).

We sought to determine the effect of Rad53 phosphorylation on Xrn1 function, and determined that the phosphorylation does not affect the inherent nuclease activity of Xrn1, nor does it appear to affect Xrn1’s interaction with any of the P-bodies proteins that we have tested. Xrn1 is also involved in rRNA processing and removal of tRNA introns (Stevens *et al.* 1991; Wu and Hopper 2014). Our Northern blot result in Figure 3A suggests that it is unlikely that Rad53 is targeting Xrn1 to alter rRNA processing. However, the effect of Rad53-dependent phosphorylation of Xrn1 on tRNA introns remains to be tested. Our attempt to study specifically the Rad53-dependent phosphorylation residues in Xrn1 was inhibited by the fact that changing 32 serines and threonines to alanines disrupted the nuclease function of Xrn1.

## Discussion

### The DDR regulates RNA metabolism through multiple substrates and pathways

Of the 54 substrates that we have identified in our screen, 24 are involved in some aspect of RNA biology. In particular, several distinct substrates in the mRNA decay pathway were identified in our screen (Figure 2A). Similarly, processing of the 35S pre-rRNA also appears to be targeted by the DDR through multiple distinct substrates (Figure 2B). Nup1, Nup2, Nup60, and Npl3 have previously been implicated in the DNA damage response and contribute to genome stability (Santos-Pereira *et al.* 2014; Ibarra and Hetzer 2015). Targeting multiple proteins in the same pathway suggests that there is a general functional rewiring of these pathways in response to DNA damage by the checkpoint.

Xrn1 is involved in key aspects of the mRNA life cycle and the cellular dynamics of mRNAs are tightly regulated. In cells, actively translating mRNAs are associated with ribosomes and the translation initiation machineries, while non-translating mRNAs are sequestered into cytoplasmic P-bodies or stress granules (Decker and Parker 2012). These messenger ribonucleoprotein (mRNP) aggregates differ in the fate of their mRNAs and in their composition, and our screen identified several proteins involved in this dynamic (Figure 2A). P-bodies typically contain mRNAs that are targeted for decay, while stress granules consist of mRNAs that are stalled or paused in translation, contain translation initiation proteins, and can reinitiate translation (Kedersha *et al.* 2005; Decker and Parker 2012). However, there is a dynamic between translating mRNAs, P-bodies, and stress granules and mRNAs can transition between these processes. In addition to Xrn1, our screen uncovered other factors involved in mRNA dynamics: Ded1, Pab1, Rpg1, and Ecm32. Gle1, in human cells, is also found to be a component of stress granules (Aditi *et al.* 2015). Ded1, Pab1, and Rgp1 are required for re-initiation of translation from stress granules. Collectively, this suggests that Rad53 is generally regulating mRNA dynamics. The Longhese lab recently showed that cells deleted for RNA processing proteins, including *xrn1∆*, are defective in processing single-stranded DNA that is necessary for checkpoint activation and suggested that this, in part, explains the DNA damage sensitivity of *xrn1∆* cells (Manfrini *et al.* 2014). Our data show that the cell directly targets Xrn1 during DNA damage.

### The functional role of Xrn1 during DNA damage

Post-translational modification is a common mode of regulation in the cell, as it quickly and transiently affects the functional role of the protein being modified. The sensitivity of *xrn1∆* cells is consistent with a model in which phosphorylation by Rad53 activates Xrn1. Xrn1 phosphorylation does not appear to affect its stability, localization, or core activity *in vitro*. It is possible that the *in vivo* effect of Xrn1 phosphorylation may not be detectable by the methods used here, or it may target specific substrates that were not examined. However, there is no evidence that Xrn1 itself has sequence preferences or specificity, and given its diverse role in processing of mRNA, rRNA, and tRNA, it is unlikely that any sequence preferences are due solely to Xrn1. However, there is precedent for Xrn1 being responsible for degradation of specific class of substrates. In response to glucose starvation, Snf1 phosphorylates the C terminus of Xrn1 and this is involved in degradation of Snf1-targeted transcripts (Braun *et al.* 2014). One of the sites of Snf1 phosphorylation, S1330, is immediately adjacent to S1329, which we identified as a Rad53 phosphosite. This suggests that phosphorylation of the C-terminus is a general mechanism for regulating Xrn1 function, and in DNA damage, Rad53 may regulate Xrn1 to promote cellular survival. In addition, human Xrn1 has also been shown to degrade initiator tRNA under heat stress and one ortholog of *Arabidopsis* Xrn1 shows some sequence specificity (Rymarquis *et al.* 2011; Watanabe *et al.* 2013). However, the mechanism for how Xrn1 is recruited to these transcripts has not been determined. Therefore, it is possible that changes in binding partners may allow Xrn1 access to specific transcripts. These binding partners are likely to include other Rad53 substrates, given the number of substrates we identified in pathways associated with RNA metabolism. Thus, it may be difficult to detect phenotypes in individual phosphosite mutants.

In summary, we have found evidence that the DDR regulates RNA metabolism in response to DNA damage. Of the 33 novel substrates identified, we verified that 12 are directly phosphorylated by Rad53 *in vitro*: Xrn1, Gcd11, Rps7b, Ded1, Cho2, Pus1, Hst1, Srv2, Set3, Snu23, Alb1, and Scp160 (Figure 2). Of these, half are involved in RNA processes: Xrn1, Ded1, and Scp160 are mRNA binding proteins; Snu23 and Alb1 are rRNA processing proteins; and Pus1 is a tRNA:pseudouridine synthase. Together, these data suggest a concerted effort on the part of the DNA damage checkpoint pathway to alter gene expression post-transcriptionally.
